# Implications of mitochondrial network organization in mitochondrial stress signalling in NARP cybrid and Rho0 cells

**DOI:** 10.1038/s41598-017-14964-y

**Published:** 2017-11-01

**Authors:** Jarosław Walczak, Małgorzata Partyka, Jerzy Duszyński, Joanna Szczepanowska

**Affiliations:** Laboratory of Bioenergetics and Biomembranes, Department of Biochemistry, Nencki Institute of Experimental Biology, Polish Academy of Science, 3 Pasteur St, 02-993 Warsaw, Poland

## Abstract

Mitochondrial dysfunctions lead to the generation of signalling mediators that influence the fate of that organelle. Mitochondrial dynamics and their positioning within the cell are important elements of mitochondria-nucleus communication. The aim of this project was to examine whether mitochondrial shape, distribution and fusion/fission proteins are involved in the mitochondrial stress response in a cellular model subjected to specifically designed chronic mitochondrial stress: WT human osteosarcoma cells as controls, NARP cybrid cells as mild chronic stress and Rho0 as severe chronic stress. We characterized mitochondrial distribution in these cells using confocal microscopy and evaluated the level of proteins directly involved in the mitochondrial dynamics and their regulation. We found that the organization of mitochondria within the cell is correlated with changes in the levels of proteins involved in mitochondrial dynamics and proteins responsible for regulation of this process. Induction of the autophagy/mitophagy process, which is crucial for cellular homeostasis under stress conditions was also shown. It seems that mitochondrial shape and organization within the cell are implicated in retrograde signalling in chronic mitochondrial stress.

## Introduction

Impairment of mitochondrial functioning routinely generates signalling mediators that may determine the fate of this organelle and stress signals can stimulate cellular adaptation. Mitochondria have the capacity to communicate with the nucleus through structural changes in the organelle itself and via changes in fusion/fission homeostasis in a dynamic mitochondrial network. It appears that the morphology of mitochondria is an element in mitochondria-nuclear communications, especially during mitochondrial stress^1^.

Mitochondrial dynamics control mitochondrial shape, which can influence rates of respiration and calcium homeostasis and transport of the organelle^2,3^. Fusion and fission processes are controlled by multiple proteins belonging to the family of large GTPases that mediate the remodelling of the outer and inner mitochondrial membranes. Mitofusin1/2 (Mfn1/2) and Opa1 control fusion, whereas dynamin-related protein 1 (Drp1), fission 1 (Fis1), mitochondrial fission factor (Mff) and mitochondrial dynamic proteins (49 and 51 kDa) (MiD49/51) mediate the mitochondrial fission process^4,5^. Studies in systems ranging from yeast to mammalian cells have demonstrated that upon cellular activation, Drp1 translocates from the cytosol to the outer mitochondrial membrane and binds to Fis1 or Mff^6^. Drp1 then oligomerizes, and its GTPase activity increases, which results in constriction of the mitochondria at scission sites and drives mitochondrial fission^7^. Together, Drp1, Mfn1, Mfn2, and Opa1 with their respective (known and unknown) adaptor/interacting proteins work to maintain proper balance between fission and fusion^8^. Defects in mitochondrial dynamics-fission/fusion processes can lead to changes in mitochondrial trafficking and organelle communication within the cell and alterations of mitochondrial quality control. Loss of the mitochondrial fission-fusion balance is associated with many pathological processes, including mitochondrial stress, cellular senescence, and neuronal injury, as well as a number of diseases^9^.

The aim of this project was to examine whether (or how) mitochondrial shape, distribution and fusion/fission proteins are involved in the mitochondrial stress response in a cellular model subjected to specifically designed chronic mitochondrial stress.

For comparison, 3 human osteosarcoma cell lines were investigated: 1. wild-type (WT) cells; 2. cybrids with 98% heteroplasmy with point mutations in ATPase subunit 6 in the mitochondrial DNA (mtDNA); and 3. Rho0 cells lacking mtDNA. The mtDNA point mutation T8993G for Leu 156 Arg results in potent inhibition of ATP synthase subunit 6. When the mutation load is between 70% and 90%, it causes a pathological syndrome involving neurological muscle weakness, ataxia, and retinitis pigmentosa - NARP. Previously, we found that in NARP cybrid and Rho0 osteosarcoma cells, the mitochondrial network is disorganized, and the observed changes include depolarization of membrane potential and ATP level (Rho0), alternations in Ca^2+^ fluxes^10,11^ and increased oxidative stress^12^. We also shown, how different stress signals promote the cellular adaptations, probably through retrograde signalling, posttranslational modifications or other mechanisms^10–14^. One of the pro-survival, important mechanism, which is activated in cells with mitochondrial stress and compensate for the respiratory deficiency is mitochondrial biogenesis. We showed and analysed activation of many regulatory factors of mitochondrial biogenesis in NARP and Rho0 osteosarcoma cells^13^. We investigated the total mitochondrial mass, the level of representative subunits of respiratory chain complexes and ATPase and non-respiratory mitochondrial proteins such as mtHsp70 (that facilitates protein import into mitochondrial matrix), uncoupling protein UCP3 (can attenuate mitochondrial ROS production) and electron carrier cytochrome C. The level and activity of transcription factors NRF1, NRF2, TFAM and PGC-1 alfa that regulate expression of genes encoding mitochondrial proteins were also studied. We did not observe changes in the level of PGC1α (coactivator of transcription factors of NRF1 and NRF2) in NARP and Rho0 cells in comparison to control cells, however the level of NRF1 and activity of NRF2 were higher than in control cells^12,13^. All this study show that chronic mitochondrial stress caused by mtDNA point mutation-NARP and loss of mtDNA - Rho0, results in induction of mitochondrial biogenesis by triggering distinct adaptive changes in the profile of mitochondrial proteins and their regulatory transcription factors irrespective of nuclear genome.

Because the mitochondrial morphology and fission/fusion processes are also elements of quality control pathways and are implicated in the removal of damaged mitochondria through the autophagy pathway, we investigated the presence of autophagy in NARP and Rho0 cells as well.

We found that the levels of fission proteins are altered in NARP and Rho0 cells, with Drp1 levels being lower, while Fis1 and Mff levels are higher in comparison with WT control cells. The level of the Mfn2 protein, involved in the fission process, is higher in NARP and Rho0 cells. Opa1 is also activated by increased levels of the Oma1 protease. We also observed changes in the level of autophagy markers in these cells.

Taking all of these results together, we suggest that the shape and distribution (dynamics) of mitochondria are involved in the retrograde signalling pathway within the cell and that transitions of mitochondrial morphology network within the cell are important responses to chronic mitochondrial stress signalling. It appears that the cooperation of fusion/fission proteins in the chronic mitochondrial stress response pathway is more complex than under acute mitochondrial stress. Moreover, our findings reveal the full spectrum of the characteristics of mitochondrial dynamics in mitochondrial diseases associated with mtDNA mutations for the first time.

## Results

### Cellular morphology and shape of mitochondria under chronic mitochondrial stress

**Figure 1:**
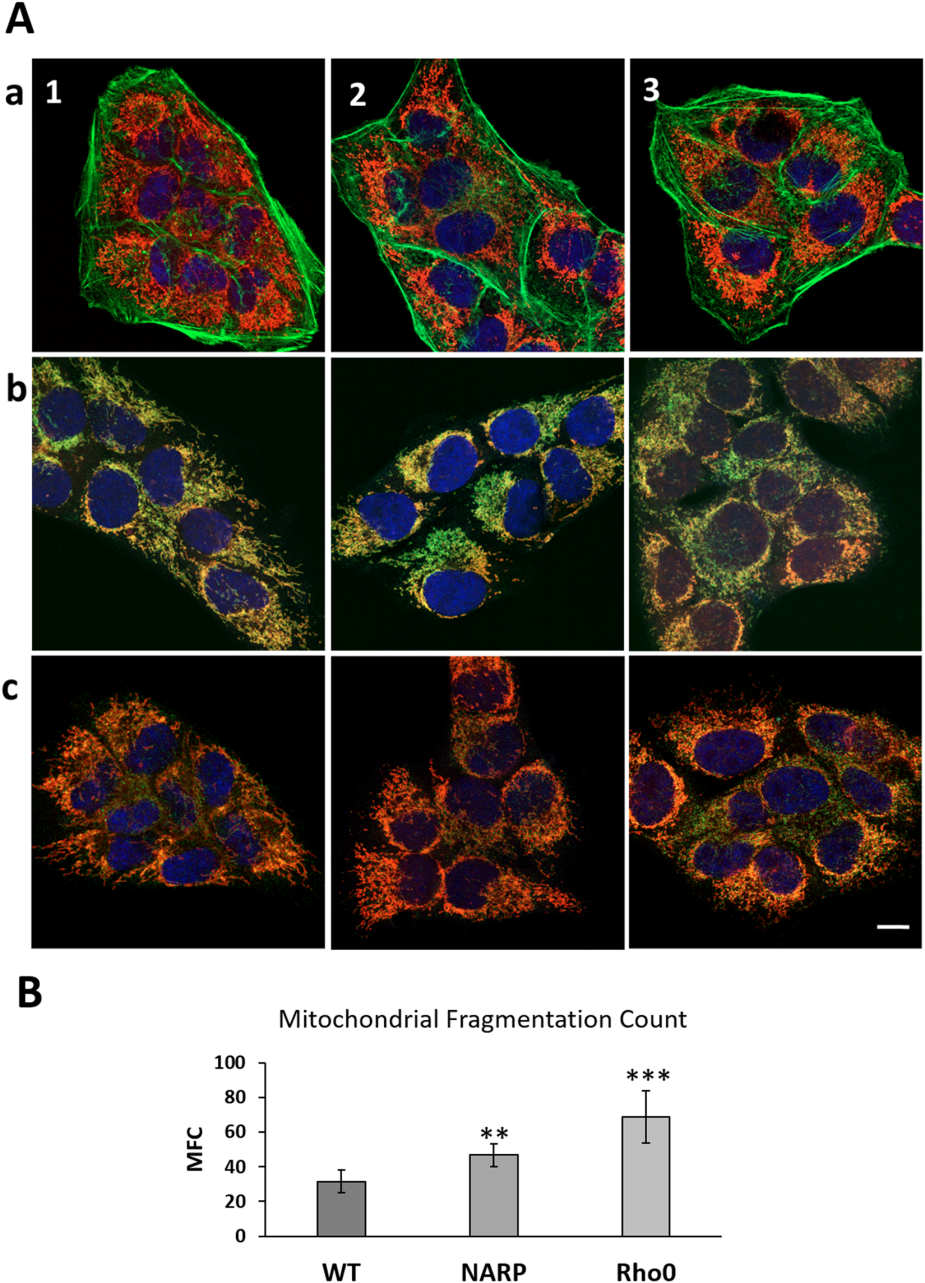
(**A**) Organisation of mitochondrial network in (1) WT, (2) NARP and (3) Rho0 cells, (a) together with actin filaments –green fluorescence, (b) Fis1 – green fluorescence (c) Drp1 - green fluorescence. Mitochondria – red fluorescence, nucleus- blue fluorescence. Scale bar represents 10 μm. (**B**) Mitochondrial Fragmentation Count, images of mitochondrial network were analysed by particle count subroutine using ImageJ software according to procedure described by Rahman *et al*.^15^. The number of counted mitochondrial fragments were normalized to the total mitochondrial area (in pixels).

**Figure 2:**
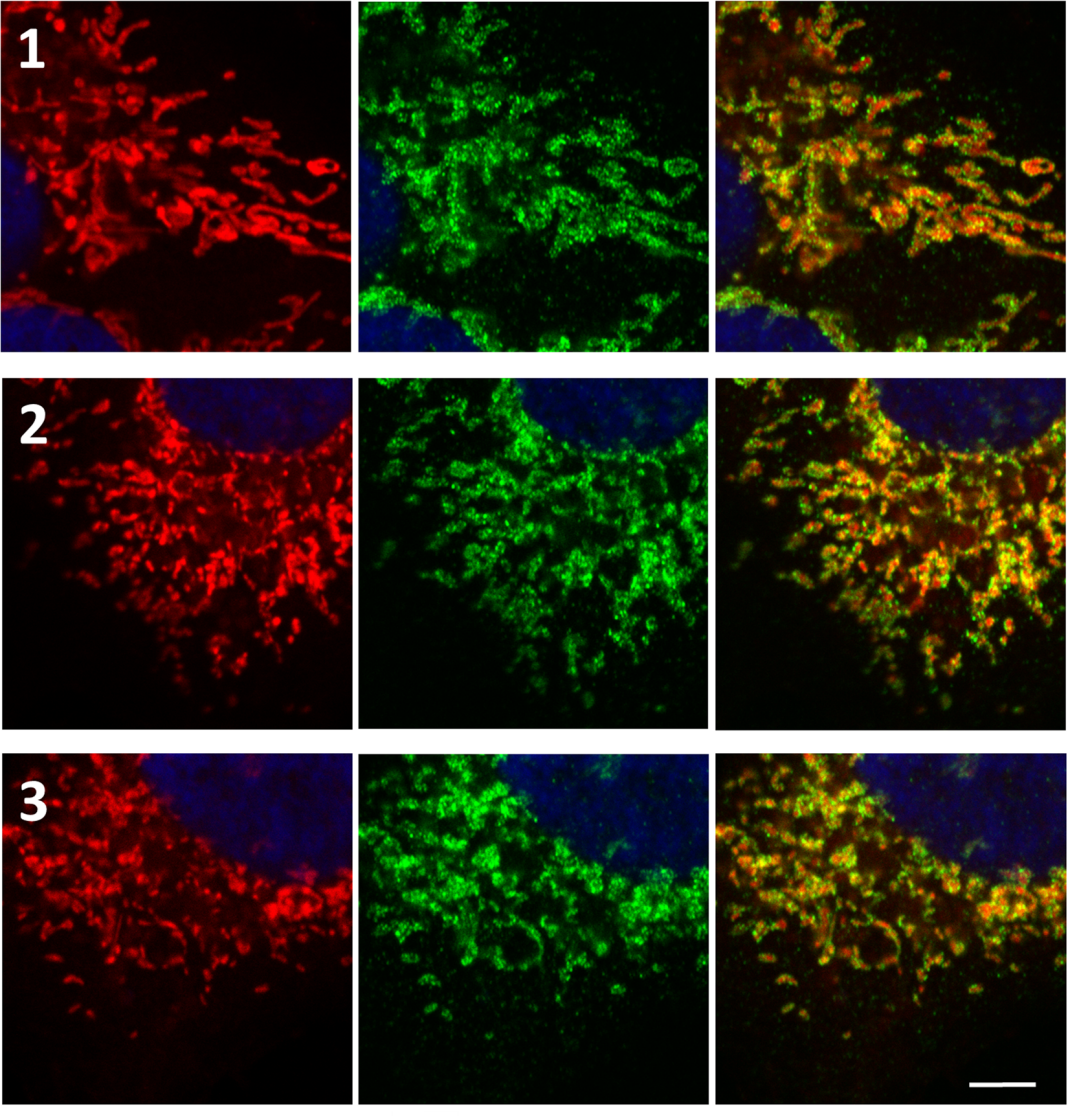
Localization of Fis1 protein in (1) WT, (2) NARP and (3) Rho0 cells. Localization of Fis1 protein in Fis1 – green fluorescence, mitochondria – red fluorescence, nucleus – blue fluorescence. Scale bar represents 2.5 μm.

**Figure 3:**
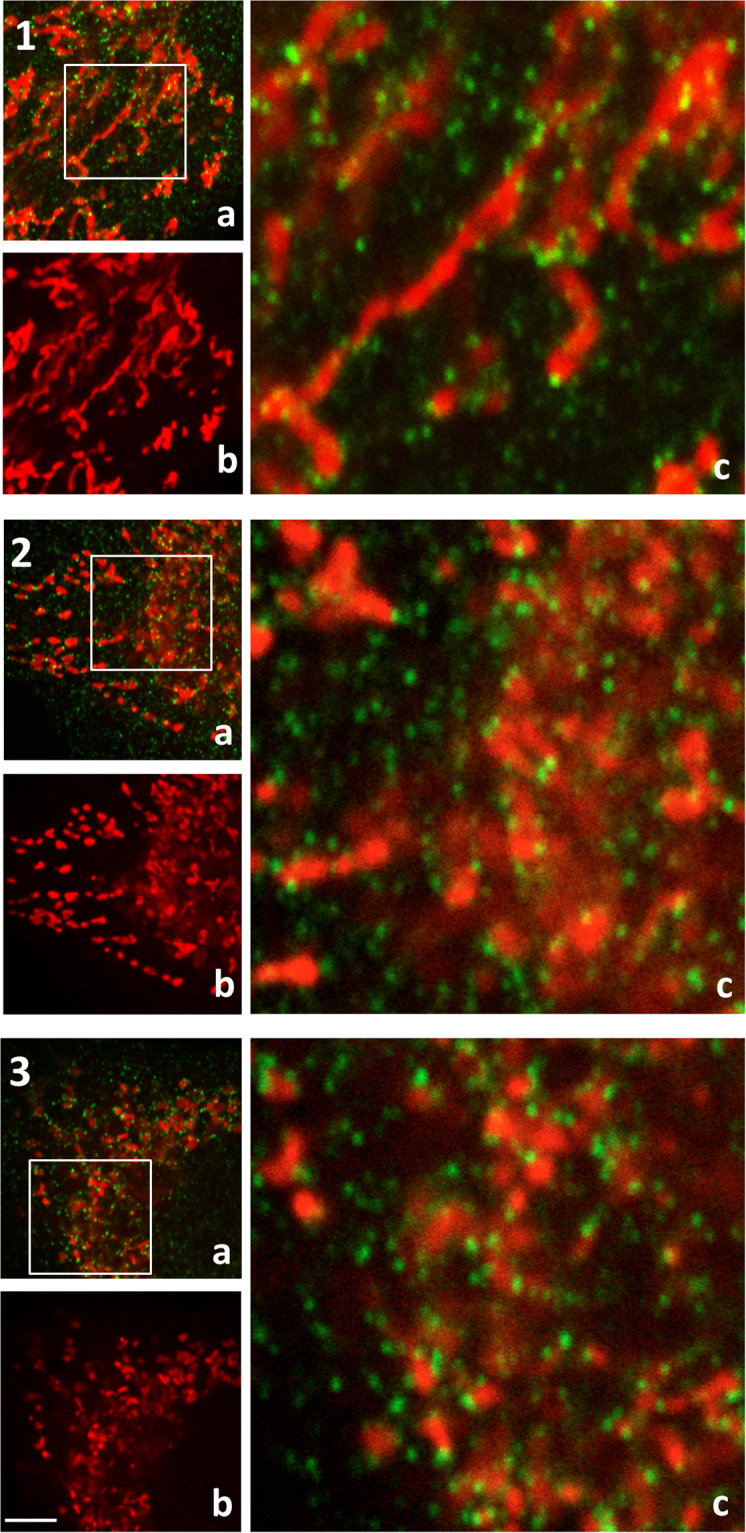
Visualisation of mitochondrial organisation and Drp1 distribution in (1) WT, (2) NARP and (3) Rho0 cells. Mitochondria – red fluorescence, Drp1 – green fluorescence: a) merge fluorescence, (b) only red fluorescence (c) small squares represent × 4.5 zoom images of a. Scale bar represents 2.5 μm.

We previously showed that mitochondria in WT osteosarcoma cells exhibit an elongated shape and form tubular network-like structures throughout the cell body, whereas in NARP cells, the shape of the mitochondria varies from tubular to rounded to punctate, and the mitochondria are more fragmented in the peripheral cell body. In Rho0 cells, the mitochondria are fragmented and form short tubules and spheres^10^. These observations were confirmed by quantification of mitochondrial network fragmentation according to the procedure described by Rehman *et al*.^15^. The mitochondrial fragmentation count (MCF) was significantly higher in NARP and Rho0 cells in comparison to WT, indicating for more fragmented mitochondrial network in these cells (Fig. 1B). Here, we sought to show the cellular morphology (Fig. 1a, actin filaments stain) and compare mitochondrial organization within the cell, especially with the localization and distribution of fission proteins Fis1 and Drp1 (Figs 1–3). As seen in Figs 1b and 2, the Fis1 protein is uniformly distributed in the outer mitochondrial membrane and the fluorescence of Fis1 determines mitochondrial organization within the cell.

The majority of Drp1 oligomers/clusters engaged in the fragmentation and disconnection of mitochondria are distributed at the edge of fragmented mitochondria, as shown in Figs 1c and 3. Because the mitochondria are distinctly fragmented in Rho0 cells, we also observe more clusters of Drp1 in these cells. The localization of these clusters defines the length of fragments of mitochondria subjected to fission. The WT mitochondrial fragments (designed by Drp1) are longer than those observed in Rho0 and NARP cells (Fig. 3, enlarged image).

### Proteins involved in the fission/fusion process

To examine the involvement of mitochondrial dynamics in mitochondrial stress signalling, we quantified the levels of these proteins.

### Fission

**Figure 4:**
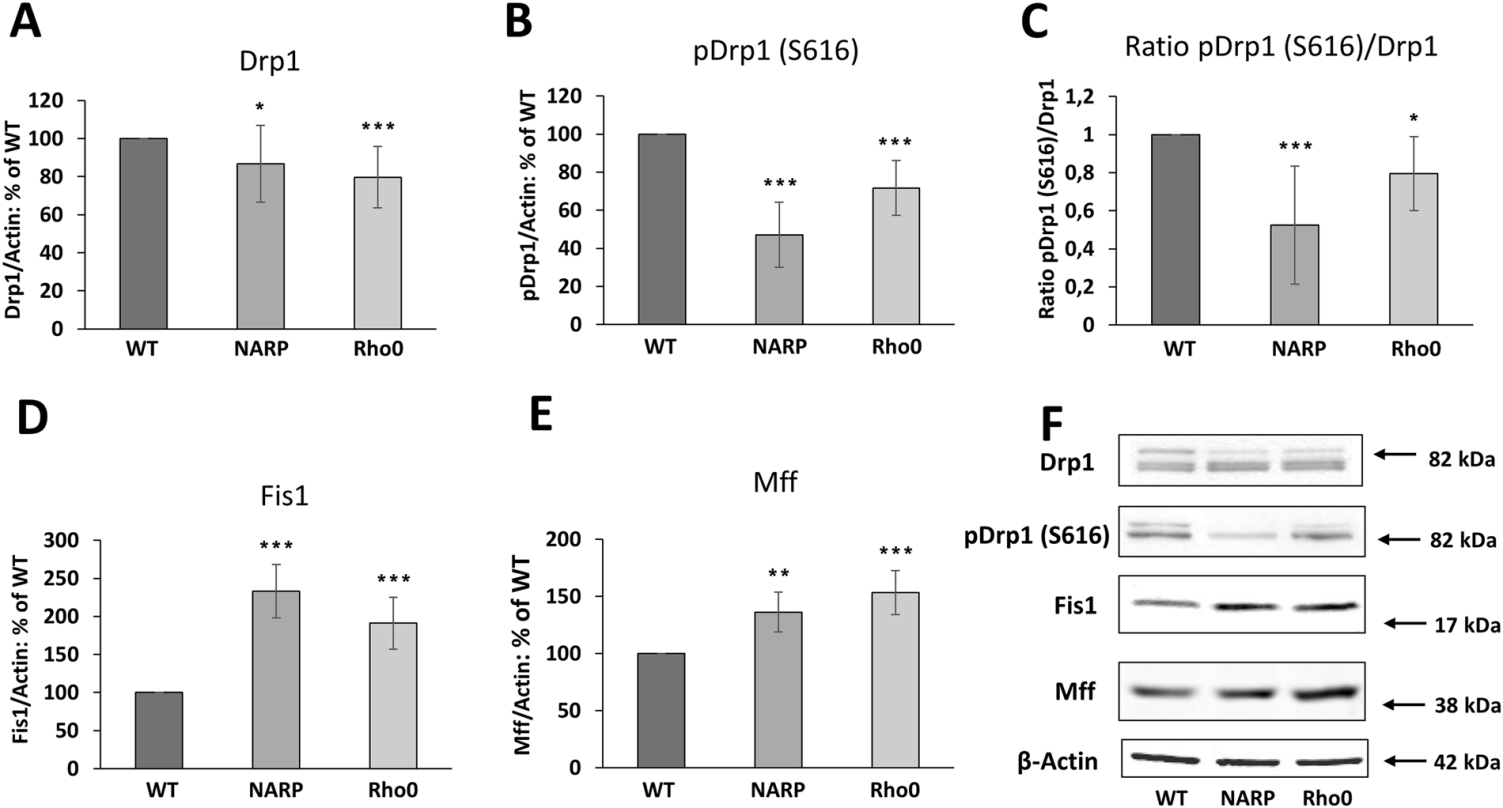
Levels of key fission factors. (**A**) Drp1 protein; (**B**) Drp1 protein phosphorylated at *Ser* 616 (Drp1-P); (**C**) ratio of Drp1-P/Drp1-total; (**D**) Fis1 protein; (**E**) Mff protein. Full-length blots are presented in Supplementary Figure 4. The values in the graphs represent the means ± SD from at least 3 independent experiments, and representative WB images are shown (F); β-actin was used as the loading control. The asterisks indicate values that are significantly different from the controls (WT cells): *P < 0.05; **P < 0.01, ***P < 0.001.

Because on the Fig. 3 we mainly observed the clustered (oligomerized) form of DRP1 under confocal microscopy and this method does not allow quantify assessment, we wanted to evaluate total amount of Drp1 (oligomerized and not oligomerized form) by WB analysis. We found that the total level of Drp1 was slightly lower in NARP and Rho0 cells than in WT, by 13 ± 20% (*P* < 0.02, *n* = 5) and 20 ± 16% (*P* < 0.001, *n* = 5), respectively (Fig. 4A). As the phosphorylation of Drp1 regulates mitochondrial fission, we examined Drp1 phosphorylation at Ser-616 (pS616-Drp1), which enhances its activity for recruitment to mitochondria. The level of the phosphorylated form of the Drp1 protein was significantly lower in NARP and Rho0 cells than in the WT, by 53 ± 17% in NARP cells and 28 ± 14% (*P* < 0.001, *n* = 5) in Rho0 cells (Fig. 4B). The ratio of Drp1-P/Drp1-total was also lower in NARP and Rho0 cells versus control-WT by 0.48 ± 0.31 (P < 0.001; n = 5) and 0.21 ± 0.13 *(P* < 0.0*3; n* = *5)*, respectively (Fig. 4C).

Four receptors that recruit Drp1 to mitochondria have been identified, but the main proteins involved in this process are Fis1 and Mff. Fis1, the first identified candidate receptor, plays a minor role in Drp1 recruitment^6,16^, and the most recent findings suggest that it plays a role in mitophagy^17^. It was recently demonstrated that the Mff protein mainly recruits cytosolic Drp1 to sites of ensuring fission and that Mff differentially regulates the activity of various isoforms of Drp1. We observed higher levels of Fis1 in both NARP and Rho0 cells, of 133 ± 35% and 91 ± 34% (*P* < *0*.001, *n* = 4), respectively, compared with WT (Fig. 4D). Similarly, the levels of Mff were also higher in NARP and Rho0 cells, by 36 ± 17% (*P* < 0.002, *n* = 3) and 53 ± 19% (*P* < 0.001, *n* = 3) in comparison with WT (Fig. 4E).

### Fusion

**Figure 5:**
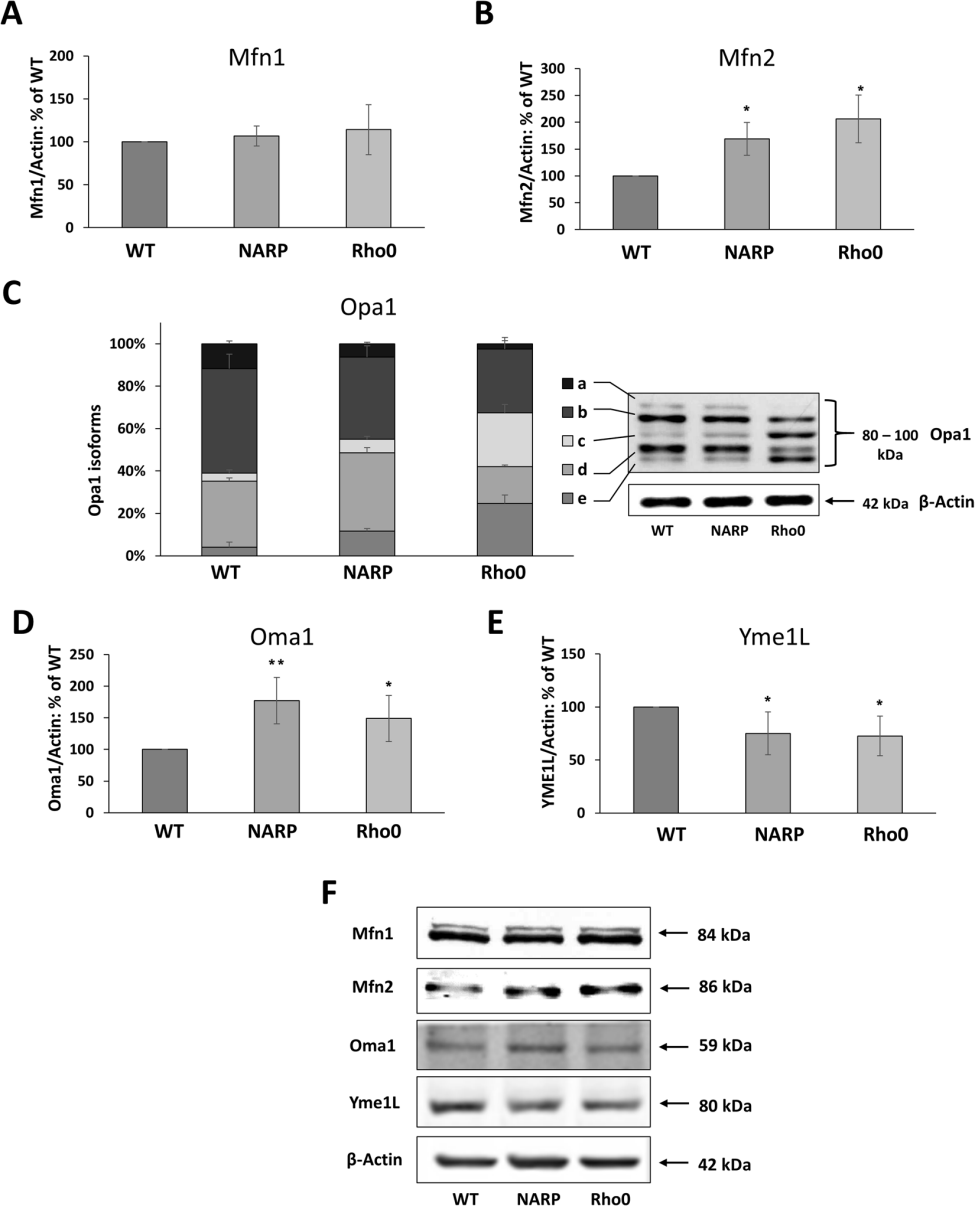
Levels of key fusion factors and mitochondrial proteases involved in the processing of the Opa1 protein. (**A**) Mfn1 protein; (**B**) Mfn2 protein; (**C**) the percentage of particular Opa1 forms (indicated as *a*–*e*); (**D**) levels of the mitochondrial protease Oma1 responsible for the proteolytic cleavage of Opa1 at the S1 site and generation of the *c* and *e* forms; (**E**) levels of the mitochondrial protease Yme1L responsible for the proteolytic cleavage of the Opa1 protein at the S2 site and generation of the *e* forms^63,64^. The values on the graphs represent the means ± SD from at least 3 independent experiments, and representative WB images are shown (**F**); β-actin was used as the loading control. Full-length blots are presented in Supplementary Figure 5. The asterisks indicate values that are significantly different from the controls (WT cells): *P < 0.05; **P < 0.01, ***P < 0.001.

Mitochondrial fusion requires fusion of both the outer and inner membranes. At the outer mitochondrial membrane, this process is directed by two proteins, mitofusin 1 (Mfn1) and mitofusin 2 (Mfn2). We did not find any statistically significant differences in the levels of Mfn1 between WT, NARP and Rho0 cells, while the levels of Mfn2 were increased in NARP and Rho0 cells by 70 ± 30% *(P* < 0.0*2; n* = *3)* and 106 ± 44% *(P* < 0.*02; n* = *3)*, respectively, in comparison with WT (Fig. 5A,B,F).

The Opa1 protein mediates the fusion of the mitochondrial inner membrane (IM) and maintains cristae morphology^18^. Long forms of Opa1 mediate mitochondrial fusion, whereas short forms of Opa1 promote the fragmentation of mitochondria, which indicates that they are associated with fission. We evaluated the total level of this protein and the levels of the long and short forms. The total level of the Opa1 protein was slightly lower in NARP cells than in WT, by 14 ± 10% (*P* < *0*.003, *n* = 4) (Fig. 5C).

It should be noted that Opa1 is regulated in the specific manner by alternative mRNA splicing and proteolysis at S1 and S2, driven by the mitochondrial proteases Yme1L and Oma1. Opa1 isoforms migrate as a complex mixture of at least five bands (*a*–*e*). The proteolytic processing of the long (L-Opa1) forms *a* and *b* at the S1 site leads to generation of the short forms (S-Opa1) *c* and *e*, while processing at the S2 site results in the formation of the short form *d* ^19^. The percentage of the long (*a* and *b*) forms of total Opa1 was lower in NARP and Rho0 cells, where it reached 45 ± 2% (*P* < 0.002, *n* = 4) and 33 ± 7% (*P* < 0.002, *n* = 4), in comparison with 61 ± 3% in WT. Conversely, the percentage of the short isoforms of Opa1 (*a*, *b* and *c*) was increased in NARP and Rho0 cells to 55 ± 2% (*P* < 0.002, *n* = 4) and 67 ± 7% (*P* < 0.002, *n* = 4), respectively (Fig. 5C).

Two proteases are involved in and required for Opa1 formation: the Oma1 protease is required for S1 cleavage, which occurs at basal levels but can be dramatically induced by the depolarization of mitochondria, and the Yme1L protease is involved in cleavage at the S2 site. We examined the presence and levels of these proteases in cells. We observed decreases in the level of the Yme1L mitochondrial protease in both NARP and Rho0 cells in comparison with WT of 25 ± 20% (*P* < 0.05, *n* = 3) and 27 ± 17% (*P* < 0.03, *n* = 3), respectively (Fig. 5E,F), while the level of the Oma1 protease was increased by 77 ± 37% (*P* < 0.01, *n* = 4) and 49 ± 36% (*P* < 0.04, *n* = 4) compared with WT (Fig. 5D,F). The cleavage of both the *a* and *b* isoforms of Opa1 was higher in both NARP and Rho0 cells but was more pronounced in the latter. The levels of specific products/forms of proteolytic cleavage of Opa1 (*a*, *b* and *c* bands) were different in NARP and Rho0 cells in comparison with WT. In NARP cells, we observed a slight increase in Opa1 bands *c* and *d* and a greater, three-fold increase in the *e* band in comparison with WT cells. In Rho0 cells, we observed a much higher, six-fold increase in the level of the *c* and *e* bands in comparison with WT cells, while the level of the *d* band was two times lower than in WT (Table 1).

**Table 1:** Evaluation and comparison of Opa1 processed bands. The specific bands (*a*, *b*, *c*, *d* and *e*) were densitometrically evaluated, and the values are presented as the percentage of total Opa1 ± S.D.

Opa1 forms (%)	WT	NARP	Rho0
a	11.73 ± 1.35	6.31 ± 0.66 *P* < 0.001	2.44 ± 1.49 *P* < 0.001
b	49.34 ± 6.88	38.78 ± 5.42 *P* < 0.02	30.19 ± 5.37 *P* < 0.02
c	3.73 ± 1.65	6.38 ± 1.48 *P* = 0.1	25.32 ± 4.07 *P* < 0.001
d	31.16 ± 1.55	36.88 ± 2.55 *P* < 0.02	17.34 ± 0.68 *P* < 0.001
e	4.04 ± 2.43	11.64 ± 1.28 *P* < 0.01	24.71 ± 3.9 *P* < 0.001

### Autophagy under chronic mitochondrial stress

Autophagy/mitophagy is an important mitochondrial quality control mechanism that eliminates damaged mitochondria. Specifically, mitochondrial fission is an essential requirement for mitophagy. The autophagy machinery can target specific substrates for degradation depending on cellular requirements in specific contexts, such as mitochondrial stress.

Autophagy is mediated in a coordinated manner by various proteins, such as Beclin1 and microtubule-associated protein 1 light chain 3 (LC3). Beclin 1 is a part of the class III PI3K complex that participates in autophagosome formation, mediating the localization of autophagy proteins to the autophagosomal membrane. LC3 is converted from the cytoplasmic form, LC3-I, to the autophagosome-bound form, LC3-II, and is considered a marker of autophagy activation. LC3-I (18 kDa) is the cytoplasmic protein. The lipidated form of the protein, LC3-II (16 kDa) is recruited to autophagosomes. The LC3-II/LC3-I ratio is used as an index of autophagy^20,21^.

**Figure 6:**
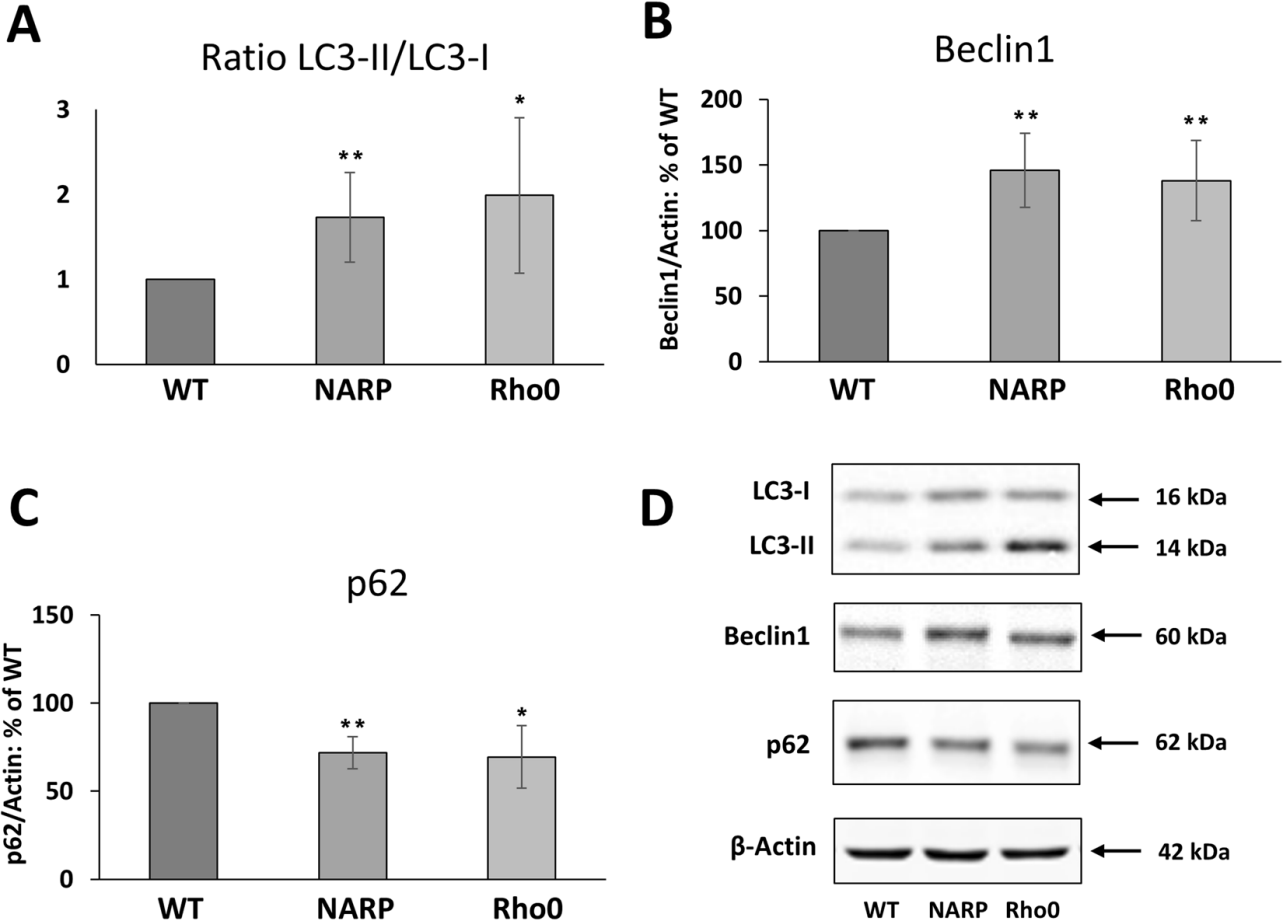
Evaluation of autophagy markers. (**A**) Ratio of LC3 II/LC3 I showing the conversion rate of LC3 I to LC3 II; (**B**) levels of the Beclin 1 protein; (**C**) levels of the p62 protein. The values in the graphs represent the means ± SD from at least 3 independent experiments, and representative WB images are shown (**D**); β-actin was used as the loading control. Full-length blots are presented in Supplementary Figure 6. The asterisks indicate values that are significantly different from the controls (WT cells): *P < 0.05; **P < 0.01, ***P < 0.001.

We evaluated autophagy markers in the investigated cell lines. The LC3 II/LC3 I ratio, which indicates the conversion rate of LC3 I to LC3 II, was higher in both NARP and Rho0 cells in comparison with WT, by 0.73 ± 0.52 (*P* < 0.004, *n* = 4) and 0.99 ± 0.91 (*P* < 0.02, *n* = 4), respectively (Fig. 6A,D). The level of the Beclin1 protein was also increased in NARP and Rho0 cells, by 46 ± 28% (*P* < 0.002, *n* = 4) and 38 ± 30% (*P* < 0.003, *n* = 4), respectively (Fig. 6B,D).

Because increasing evidence correlates p62 with the clustering of damaged mitochondria^22^, we evaluated the level of this protein. We found that the level of p62 was lower 28% ± 9% *(P* < 0.002, *n* = *4)* in NARP cells and 31% ± 17% *(P* < 0.02, *n* = *4)* in Rho0 cells in comparison with WT (Fig. 6C,D). To examine the role of p62 in autophagy in cells with mitochondrial dysfunction we compared p62 level in starved cells (induction of autophagy) and in cells treated with inhibitor of this process – chloroquine (Fig. 7 in Supplementary Data)^21^. Analysis indicate that after 2 hours of starvation the level of p62 decreased in all treated cells but there were no statistically significant differences between NARP, Rho0 and WT cells. Co- treatment (starvation and CQ) did not lead to increase of p62 level but it caused even decrease of p62 in Rho0 cells by 33% ± 10% *(P* < 0.001, *n* = *3)* (Supplementary Data Fig. 7A). Because the inhibition of autophagy by chloroquine did not lead to accumulation of p62 in Rho0 cells we extended the incubation time with CQ to 8 hours. The starvation of Rho0 cells for 8 hours was not possible as these cells are extremely sensitive for such conditions and would not survive. The level of p62 in WT and NARP cells was not changed, but in Rho0 cells decreased by 15% ± 6% (*P* < 0.05, *n* = 3) (Supplementary Data Fig. 7B). These data may suggest that in Rho0 cells the autophagosome clearance may be impaired or autophagy process is not as dynamic (plastic) as in control cells. Although we did not see accumulation of LC3II form in response to 2 hours starvation in PBS, the LC3 turnover was increased in all investigated cell lines upon the 2 hours treatment of chloroquine (Supplementary Data Fig. 7A) and this effect was stronger after elongated exposure to CQ (Supplementary Data Fig. 7B).This results may indicate that in Rho0 cells (strong mitochondrial stress - respiratory chain subunits deficiency) the autophagy process (also process of removing of damaged mitochondria) is essential for Rho0 cells survival and may also indicate that this protein may plays other necessary role in cell after adaptation to stress conditions.

**Figure 7:**
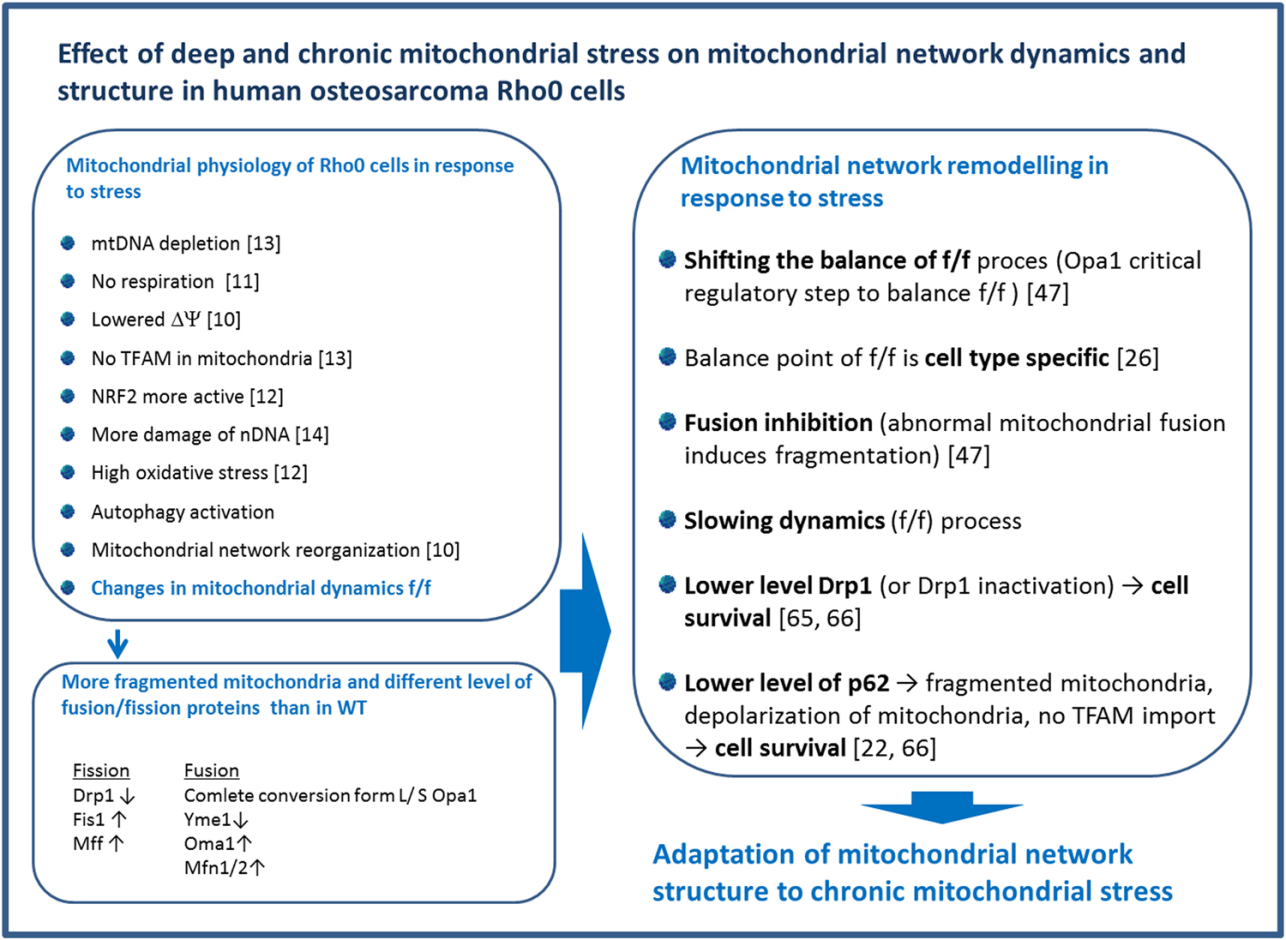
Scheme summarizing „Implications of mitochondrial network organization in mitochondrial stress signaling in Rho0 cells” (model of deep and chronic mitochondrial stress)^22,26,47,65,66^.

## Discussion

Based on our previous findings showing that point mutations in subunit 6 of ATP synthase in mtDNA cause chronic mitochondrial stress, manifested in increased levels of ROS, elevation of cytoplasmic calcium ion levels, and alterations of the mitochondrial membrane potential and mitochondrial network organization within the cell^10–12^, we concluded that mitochondrial shape and the mitochondrial distribution might be involved in the modulation of mitochondrial stress signalling pathways. Mitochondrial dynamics (including fusion/fission and the translocation of mitochondria) are crucial to maintain cellular homeostasis, and this process plays a significant role in cellular polarity and stress responses^23^.

Therefore, we characterized the mitochondrial distribution and evaluated the levels of fusion/fission proteins involved in mitochondrial dynamics using a cellular model under two different types of chronic mitochondrial stress: NARP (intermediate) and Rho0 (high) stress.

The total level of the fission protein Drp1 was lower in NARP and Rho0 cells in comparison to WT, although a more fragmented mitochondrial network was observed especially in Rho0^10^ than in WT cells (Figs 1 and 2). This means that the total level of Drp1 is not correlated with the high level of mitochondrial fragmentation present in Rho0 cells. Not only the level of the Drp1 form phosphorylated at position 616 (which is responsible for the activation of Drp1^24,25^) was lower in these cells; the ratio of total Drp1/ Drp1-P616 was also lower in comparison with WT cells. One possibility is that in osteosarcoma cell type, additional posttranslational modification of Drp1 protein could be engaged in mitochondrial fragmentation and that functional consequence of serine phosphorylation of Drp1 could be highly cell-type dependent. It should be emphasised, that the pattern of mitochondrial proteins involved in dynamics is dependent on the cell type. The group of David Chen shown that the precise balance point between fusion and fission is also cell specific^26^. For example in endometrial cancer cells with mitochondrial stress (caused by complex I deficiency) the total level of Drp1 protein was different than in NARP and Rho0 cells^27^.

The second one explanation is that regulation of fission process may also occur at the level of the Drp1 protein recruited to the mitochondrial active fission sites. Although the total level of Drp1 was lower in NARP and Rho0 cells, the levels of Fis1 and Mff (receptor proteins that recruit Drp1 to mitochondria) were much higher in these cells, which could indicate that more labelled sites for the recruitment of Drp1 are present in the outer mitochondrial membrane. According to Macdonald *et al*.^28^, the Mff-dependent recruitment of two isoforms of Drp1 is differentially regulated. Mff is a primary receptor that recruits only the active form of Drp1 from the cytoplasm to fission sites^29^. In mouse embryonic fibroblasts, loss of Mff results in the greatest reduction of the fission process, while overexpression causes mitochondrial fragmentation, consistent with increased fission rates^6,16^. We suggest that in osteosarcoma Rho0 and NARP cells, Mff plays a principal role in Drp1 recruitment to the outer mitochondrial membrane and initiates mitochondrial fission, guiding this process towards the main cellular stress response pathways. Therefore, we were not able to detect additional spots designed for fission initiation (Figs 2 and 3 labelled Fis1 and Drp1). Guido *et al*. have shown^30^ that mitochondrial fission induces glycolytic reprograming and promotes early tumour growth. We have to remember that osteosarcoma Rho0 are cancer cells with a very severe mitochondrial defect (deep stress) therefore the pattern of fusion/fission protein could be different.

Sauvanet *et al*.^31^ showed that Rho0 yeast cells exhibit an additional possible mechanism of fragmentation of the mitochondrial network through alternation and inhibition of the mitochondrial fusion process. It is possibly that in osteosarcoma Rho0 cells abnormal fusion induces the fragmentation of mitochondria. Therefore, we assessed the levels of the main proteins involved in mitochondrial fusion, such as Mfn1/2 and Opa1. While the levels of Mfn1 in NARP and Rho0 cells were comparable to those in WT cells, the level of Mfn2 was significantly increased (Fig. 5B). These results were confirmed via confocal microscopy observations, in which mitochondrial fusion was found in the proximity of the nucleus. Mfn2 is not only involved in fusion of the outer mitochondrial membrane but also participates in the association of the mitochondrial outer membrane with the ER^32^ and an adaptor protein (MIRO) responsible for the transport of mitochondria^33^. It has been shown that Mfn2 deficiency induces apoptosis^34^. Therefore, increased levels of Mfn2 in Rho0 cells can prevent apoptosis in cancer osteosarcoma cells and could also participate in contact ER-mitochondria sites in fission process. We suggest that under severe chronic mitochondrial stress (Rho0), elevated levels of the Mfn2 protein may take part in the stress response pathway. Overexpression of Mfn2 is known to play a protective role in mitochondrial functioning^34^ and could partially rescue the metabolic damage in mtDNA-deficient cells^35,36^. Therefore, higher level of Mfn2 in Rho0 cells could be a result of adaptation of cells to chronic mitochondrial stress.

Another fusion-promoting protein, Opa1, mediates the fusion of the mitochondrial inner membrane and maintains cristae morphology^33–39^. The activity of Opa1 is regulated in response to mitochondrial stress^36,40–42^. Stress-induced Opa1 processing inhibits fusion, leads to mitochondrial fragmentation, and affects the segregation of damaged mitochondria^43^. In physiological conditions, the proteolytic cleavage of long isoforms of Opa1 (L-Opa1) results in balanced accumulation of long and short Opa1 (S-Opa1) forms^41,44,45^, which we observed in WT cells. In NARP cells, we detected a slight increase in the level of short forms, but in Rho0 cells, the balance of the long and short forms was completely altered, favouring mitochondrial fission. This finding is in agreement with our previous results showing a significantly lower mitochondrial membrane potential in Rho0 cells^11^, possibly leading to induction of the complete conversion of L-Opa1 into S-Opa1, as shown previously^41–43,45,46^. Proteolytic processing of Opa1 plays critical regulatory role in the balance between mitochondrial fusion and fission^47^. Disruption of the fusion process leads to a loss of mitochondrial membrane potential and decreases cellular respiration (distinct features characteristic for Rho0 cells)^48^.

Opa1 is excessively processed by the metalloproteinases Oma1 and Yme1L, releasing the majority of the protein from its transmembrane anchor and causing it to become incompetent for fusion^49,50^. When both proteases are lost (Oma1 or Yme1L knockout cells), Opa1 maintains the ability to fuse the inner membrane without proteolytic processing^47,51^. Mitochondrial depolarization has been proposed to lead to relocalization of Oma1 to the outer membrane, where it accumulates^19^. Because the mitochondria are mainly depolarized in Rho0 cells, we expected that Oma1 would be activated, and even quantitative analyses of Oma1 showed increased levels in NARP and Rho0 cells (Fig. 5D). According to Baker *et al*., Oma1 is constitutively active but displays strongly enhanced activity in response to various stress insults^37^. The degradation of Opa1 by Oma1 is a general cellular stress response process.

The second protease, Yme1L, regulates the processing and stability of the Opa1 protein^51^. Depletion of Yme1L leads to mitochondrial fragmentation^52^. Localization of Yme1L in the mitochondrial inner membrane space enhances its involvement in mitochondrial fission, in association with other proteins. It was also observed that when the level of Yme1L decreases, the level of Mff increases, and fission occurs. Similar correlations were found in NARP and Rho0 cells. Furthermore, Oma1 and Yme1L modulate a number of biochemical activities/pathways that are essential for mitochondrial functions, including some that are important for quality control pathways, such as autophagy/mitophagy.

Autophagy is one of the elements of the mitochondrial stress response and is generally considered a pro-survival process. Rizzuto group^53^ investigated autophagy process in human fibroblasts with point mutation in mtDNA (encoding ND5 complex I subunit) and show that mitophagy process is upregulated and that it is compensatory mechanism for survival of these cells. Scorrano group^54^ showed that mitochondrial network organization within the cell determine the cellular response to autophagy. During the early induction of autophagy the mitochondrial dynamics process is very active and fission process follows the elongation of mitochondria (in starvation induced autophagy). In chronic starvation, mitochondria are unable to elongate, they consume cytosolic ATP to maintain mitochondrial membrane potential^54^.

Because increased amounts of fragmented mitochondria and mitochondrial fission are necessary to separate healthy from defective parts of mitochondria in NARP and Rho0 cells, we expected that elimination of defective mitochondria would be induced in these cells. It has been proposed that fission acts as an autophagic checkpoint^55^. We showed that the levels of the fission proteins Fis1 and Mff are much higher in NARP and Rho0 cells than in WT cells. Depending on stress conditions, Fis1 can contribute to the orderly disposal of defective mitochondria through mitophagy^17^. Because autophagy in cell with chronic mitochondrial stress may help to survive, in Rho0 cells we observe activation of this process even after treatment with inhibitor of autophagy (Fig. 7 Supplementary Data). Distinct pathways may cooperate to regulate mitochondrial function in response to various stresses^56^.

Increasing evidence correlates p62, which is usually recruited to depolarized mitochondria, with the clustering of damaged mitochondria^22^. Depolarization of mitochondria results in a more fragmented morphology, with mitochondria showing perinuclear clustering (“mito-aggresomes”). p62 regulates mitophagy under stress and pathological conditions. The level of p62 is generally inversely correlated with autophagic degradation. After the fusion of autophagosomes with lysosomes, p62 is degraded by autophagy; therefore, the p62 level is lower in cells with activated autophagy. Inhibition of autophagosome maturation leads to the accumulation of p62^57^. Lower levels of the p62 protein in NARP and Rho0 cells (Fig. 6C) indicate that under chronic mitochondrial stress, the process of autophagy is active, which is also confirmed by a higher ratio of LC3 II/LC3I and an elevated level of Beclin 1 (Fig. 6A,B). We suggest that autophagy process accompanied in the remodelling of mitochondrial network in chronic mitochondrial stress response pathway. This is most visible in Rho0 cells, with fragmented mitochondrial network, very low mitochondrial membrane potential (about 30% of WT^11^) and very low level of p62 (decreases even after inhibition of autophagy, Supplementary Data Fig. 8). The group of Schon^58^ prove that both genetically (mtDNA mutation) and chemically induced loss of mitochondrial transmembrane potential has retrograde signaling effect on pro-mitophagic factors and endogenous autophagic machinery. Nunnari group^59^ showed that mitochondrial respiratory deficiency is a general regulator of the autophagic response.

But the role of p62 is not limited to autophagy. p62 is a multi-functional protein that acts as a signaling hub integrating various stress responses and plays critical roles in many diseases such as neurodegeneration and cancer. The role of p62 in mitochondrial dynamics, mitochondrial genome integrity and mitochondrial transcription factor TFAM import to mitochondria in p62^−/−^ tissues and cells was studied in^22^. The authors found that localization of p62 to mitochondria is required for mitochondrial biogenesis (takes part in TFAM transport to mitochondria) and mitochondrial energetics. In non-stressing conditions p62 is localized to mitochondria and its deficiency leads to mitochondrial dysfunction. Moreover, they showed that mitochondria in the absence of p62 became fragmented and suggested that the mitochondrial network rearrangements could be directly correlated with localization of p62 to mitochondria. We observed that the total level of p62 in Rho0 cells was always lower than in WT cells (regardless whether the autophagy process was activated and/or inhibited). Therefore we can suggest that low level of p62 could also influence mitochondrial network reorganization (fragmentation) in osteosarcoma Rho0 cells.

In summary, in human osteosarcoma cells subjected to chronic mitochondrial stress, remodelling of the mitochondrial network occurs. The organization of the mitochondrial network is correlated with permanent changes in the levels of proteins responsible for mitochondrial dynamics. Induction of the mitophagy/autophagy process (and lower level of p62 protein), which is crucial for mitochondrial remodelling organisation necessary in cellular homeostasis under stress conditions, was also shown. In the absence of mitochondrial turnover, oxidative damage would gradually accumulate and ultimately result in increased mitochondrial dysfunction, but in the case of the process of retrograde signalling pathway, cells adopt to chronic stress condition. In adapted cells (with very strong mitochondrial stress - Rho0, Fig. 7), probably remodelling of dynamics of mitochondrial network is correlated with the different level of proteins responsible for fusion and fission and with process of removing of damaged, malfunctioned mitochondria.

Our findings indicate that the consequences of mtDNA mutations are not limited to the OXPHOS defects (what we published previously^10–14^ and summarized in Fig. 7) but may also include alterations of mitochondrial shape, dynamics and mitochondrial turnover. Furthermore, we can say that coordinated changes in the fission/fusion homeostasis of mitochondria are a part of the complex of communications involved in retrograde signalling. These results confirm the importance of mitochondrial dynamics in retrograde signalling under chronic mitochondrial stress caused by mtDNA mutations. Moreover, remodelling of mitochondrial network organisation as a stress signal takes a part in process of cellular adaptation.

## Methods

### Chemicals and antibodies

High glucose Dulbecco’s modified Eagle’s medium (DMEM) and heat-inactivated fetal bovine serum (FBS) were from GIBCO (Grant Island, NY, USA). Trypsin, uridine, penicillin, streptomycin and Tween-20 were from Sigma Aldrich (St. Louis, MO, USA).

All other chemicals were of analytical grade

The anti-Drp1(Western Blotting (WB): 1:2500) and anti-Opa1 (WB: 1:2500) were obtained from BD Transduction Laboratories (San Diego, CA, USA), Mitofusin 1 (WB 1:2500) was from Abcam (Cambridge, UK), Mitofusin 2 (WB 1:2500), β-actin (WB: 1:50 000) and Fis1 (WB 1:1000) from Sigma Aldrich (St. Luis, MO, USA). Alexa Fluor 488 Phalloidin and DAPI were purchased from Invitrogen (Eugene, OR, USA).

Secondary antibodies labelled with horseradish peroxidase (WB: 1:5000) were from Santa Cruz Biotechnology (Santa Cruz, CA, USA), and antibody used for immunofluorescence staining was purchased from Jackson ImmunoResearch Europe, Ltd. (Newmarket, UK) and Invitrogen (Eugene, OR, USA). IRDye 680 and IRDye 800 Secondary Antibodies (1:5000) were from Li-Cor Biosciences (Bad Homburg, Germany).

### Cell lines and culture conditions

Parental human osteosarcoma cell line 143B, cybrid cell line derived from NARP skin fibroblasts 143B/206 with 98% penetration of the mtDNA T8993G mutation in ATP6 gene and Rho0 cell line lacking mitochondrial DNA^60,61^ were established and kindly provided by Dr. M. Tanaka of the Department of Gene Therapy, Gifu International Institute of Biotechnology, Japan.

In order to study mitochondrial dysfunction, human Rho0 cells have been established^60^. Rho0 cells are depleted of mtDNA by exposure to ethidium bromide, while maintaining the same nuclear genetic background as original cells, and therefore they are considered to be good model to study chronic mitochondrial stress. Cybrid cells were constructed by transfer of cytoplasts possessing mitochondria from fibroblasts of patients diagnosed with NARP syndrome with 98% heteroplasmy to Rho0 cells. Cybrid cells represent a useful model of chronic mitochondrial stress caused by dysfunction of ATPase.

The cells were cultured at 37 °C in a humidified atmosphere containing 5% CO_2_ in high glucose Dulbecco’s modified Eagle’s medium (DMEM) supplemented with 10% heat-inactivated fetal bovine serum (FBS), uridine and antibiotics: penicillin and streptomycin. All experiments were performed with cells between the 3rd and the 6th passages.

### Whole cell extracts

The cells were harvested with trypsine, washed with ice-cold PBS, sedimented by centrifugation and resuspended in the lysis buffer at 4 °C (50 mM Tris–HCl, 150 mM NaCl, 1% Triton X-100, 1% Nonidet P-40, 5 mM EDTA, protease and phosphatase inhibitors: (Protease Inhibitor Cocktail: Sigma #P8340, Phospahatase Inhibitor Cocktail: Sigma #P0044, 1 μg/ml aprotynin, 1 mM PMSF, 1 mM sodium orthovanadate and 10 mM NaF). After 20 min, cells lysates were homogenised and centrifuged at 8600 × *g* for 20 min at 4 °C. The supernatants were collected and the protein concentration was determined according to Bradford^62^. The samples were supplemented with SDS sample buffer (0.5 M Tris–HCl, 2.3% SDS, 5% mercaptoethanol (v/v), 12.5% glycerol (v/v); pH 6.8. 0,04% bromophenol blue) and subjected to further analysis.

### Immunoblotting

Equal amounts of protein were separated by SDS-PAGE, transferred to nitrocellulose membrane and blocked with 5% non-fat dry milk/TBS-0.05% Tween-20 solution or Blocking buffer (Li-Cor Bioscience, Lincoln, NE, USA). Subsequently the blots were incubated with appropriate primary and secondary antibodies and developed with a chemiluminescence detection kit (Bio-Rad Laboratories, Munich, Germany) or Odyssey Infrared Imaging System (Li-Cor Biosciences, Bad Homburg, Germany).

### Confocal microscopy

Cells seeded on glass coverslips were fixed with 4% paraformaldehyde in PBS pH 7.4 for 15 min at room temperature, rinsed with 10% FBS/PBS, and incubated for 1 h with primary antibodies in 10% FBS/PBS supplemented with 0.2% saponin. After washing three times with 10% FBS/PBS to remove unbound antibody, the cells were incubated for 1 hour with the appropriate fluorescently-conjugated secondary antibodies diluted in 10% FBS/PBS containing 0.2% saponin. Coverslips were washed three times with 10% FBS/PBS, once with PBS, and then mounted on a slide.

To visualize morphology of the cell architecture and mitochondrial network, the cells were incubated with 200 nM MitoTracker CMXRos (Invitrogen, San Diego, CA, USA) in the dark at 37 °C for 10 min. The cells were then briefly rinsed with the medium under the same conditions and fixed. CMXRos is a cationic lipophilic dye, preferentially sequestered into mitochondria and reacting with thiols of proteins in the mitochondrial matrix. Actin filaments were stained by Alexa Fluor 488 phalloidin (Invitrogen, Eugene, OR, USA).

Confocal fluorescence images and image stacks (0.3 μm) were collected using a Zeiss LSM780 confocal microscope with a 63× oil immersion objective. Images were acquired from randomly selected fields of nonconfluent cells. All images were processed and analyzed using Zeiss LSM780 software.

### Quantification of mitochondrial network fragmentation

Images acquired in confocal microscope were analyzed for the mitochondrial network fragmentation using ImageJ software (U.S. NIH, Bethesda, MD, USA) according to the procedure described by Rehman *et al*.^15^. Briefly, images were background subtracted, tresholded and binarized. Mitochondrial structures were counted with the particle counting subroutine and normalized to the total mitochondrial area (in pixels) to obtain the Mitochondrial Fragmentation Count (MCF). At least 25 randomly selected cells were imaged to calculate MFC values.

### Data analysis

The data represent means ± SD for at least three independent experiments. Statistical significance was assessed with Student’s *t*-test. Only *P* values lower than 0.05 were considered statistically significant. For the densitometric analysis, ImageJ Software (for chemiluminescence detection) or Odyssey software (for IRDye antibodies) were used.

## Electronic supplementary material

Supplementary PDF File
