# Supplementary material for: Implications of mitochondrial network organization in mitochondrial stress signalling in NARP cybrid and Rho0 cells
Source: Sci Rep. 2017 Nov 1;7:14864. doi: 10.1038/s41598-017-14964-y (PMC5665886; doi:10.1038/s41598-017-14964-y)
Supplement: Supplementary file 1 — Supplementary PDF File [file 41598_2017_14964_MOESM1_ESM.pdf]

*Supplementary Information*

# Implications of mitochondrial network organization in mitochondrial stress signalling in NARP cybrid and Rho0 cells

*Jarosław Walczak, Małgorzata Partyka, Jerzy Duszyński, Joanna Szczepanowska**

Laboratory of Bioenergetics and Biomembranes, Department of Biochemistry, Nencki Institute of Experimental Biology, Polish Academy of Science, 3 Pasteur St, 02-993 Warsaw, Poland

Address correspondence to: *Joanna Szczepanowska, PhD, 3 Pasteur St, 02-093 Warsaw, Poland.
Fax: 0048228225342; Phone: 0048225892345; E-mail: j.szczepanowska@nencki.gov.pl

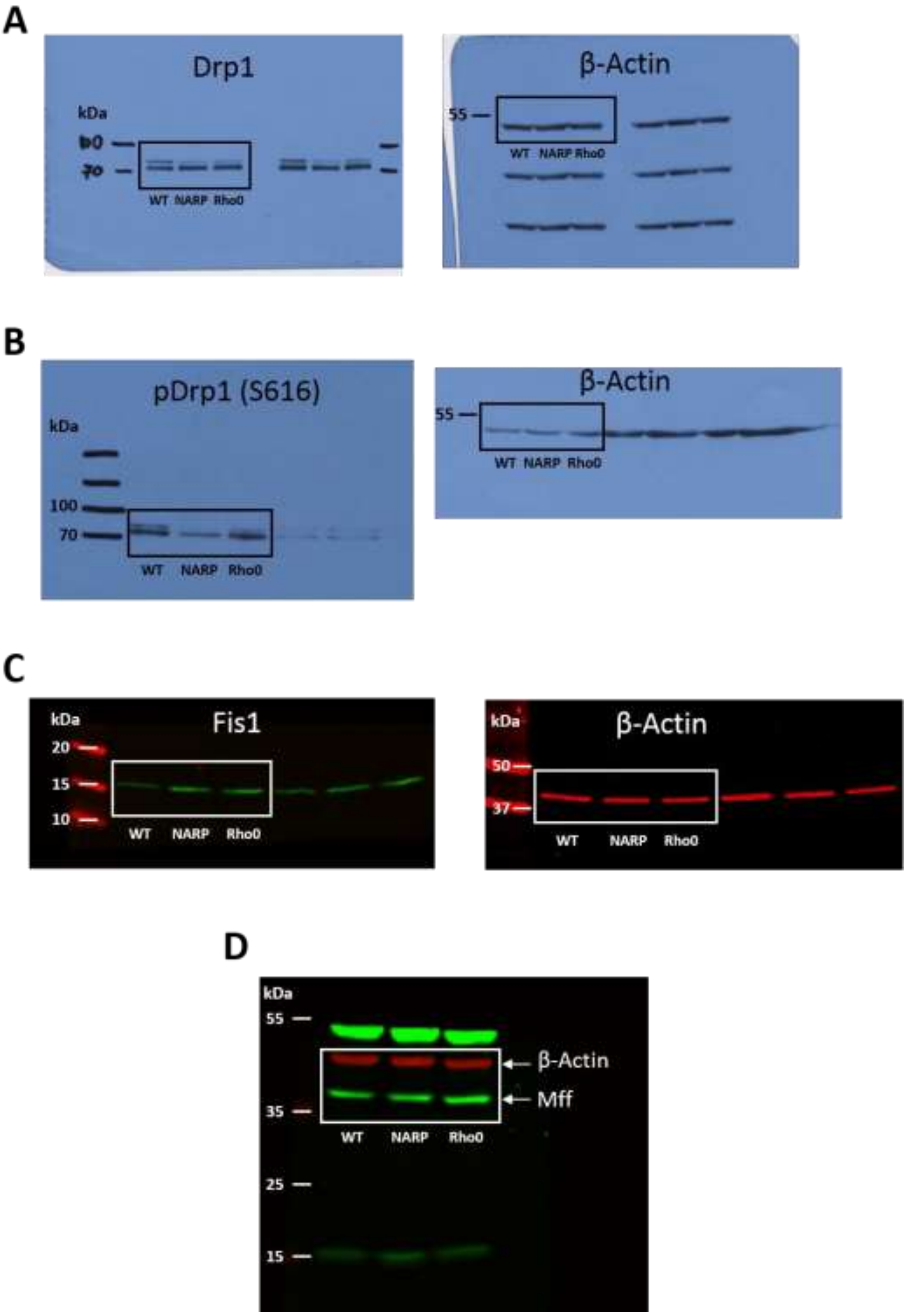


Supplementary Figure 4: full-length blots of Drp1 and β-Actin (A), pDrp1 (S616) and β-Actin (B), Fis1 and β-Actin (C), Mff and β-Actin (D). Blots A and B were developed using chemiluminescence method, blots C and D were developed with near-infrared fluorescence method (Odyssey LiCor).

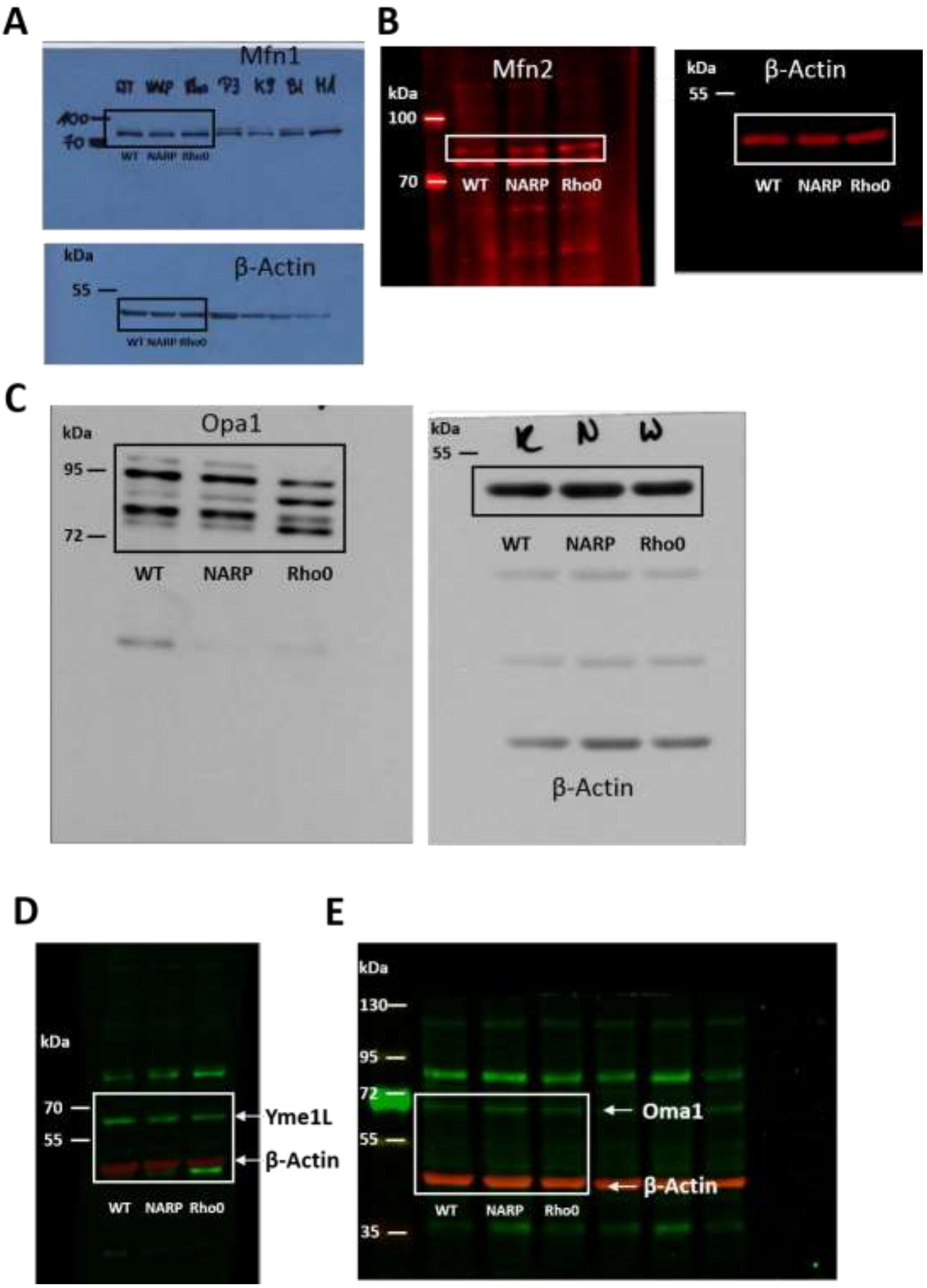


Supplementary Figure 5: full-length blots of Mfn1 and β-Actin (A), Mfn2 and β-Actin (B), Opa1 and β-Actin (C), Yme1L and β-Actin (D), Oma1 and β-Actin (E). Blots A and C were developed using chemiluminescence method, blots B, D and E were developed with near-infrared fluorescence method (Odyssey LiCor).

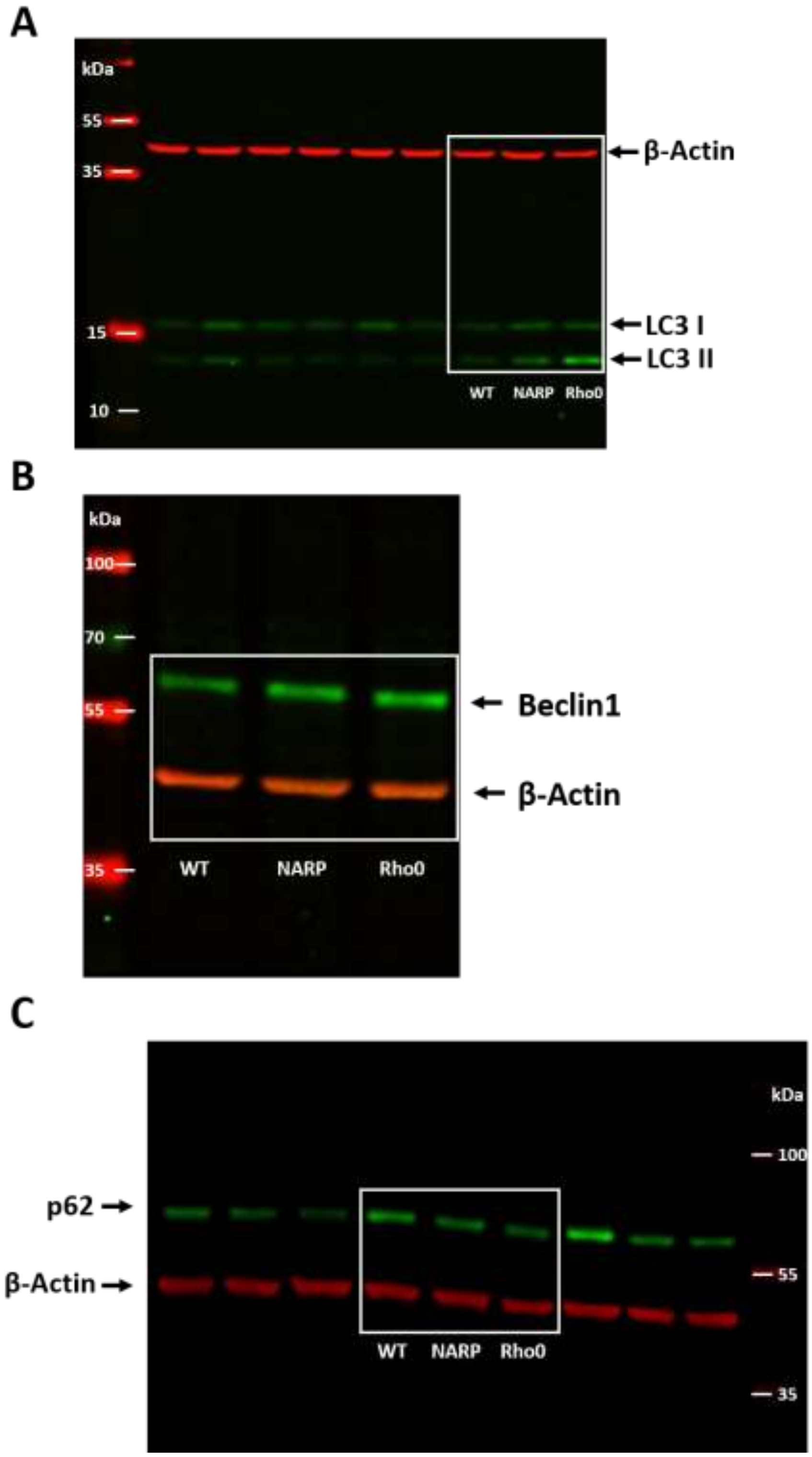


Supplementary Figure 6: full-length blots of LC3 I/II and β-Actin (A), Beclin1 and β-Actin (B), p62 and β-Actin (C). Blots were developed with near-infrared fluorescence method (Odyssey LiCor).

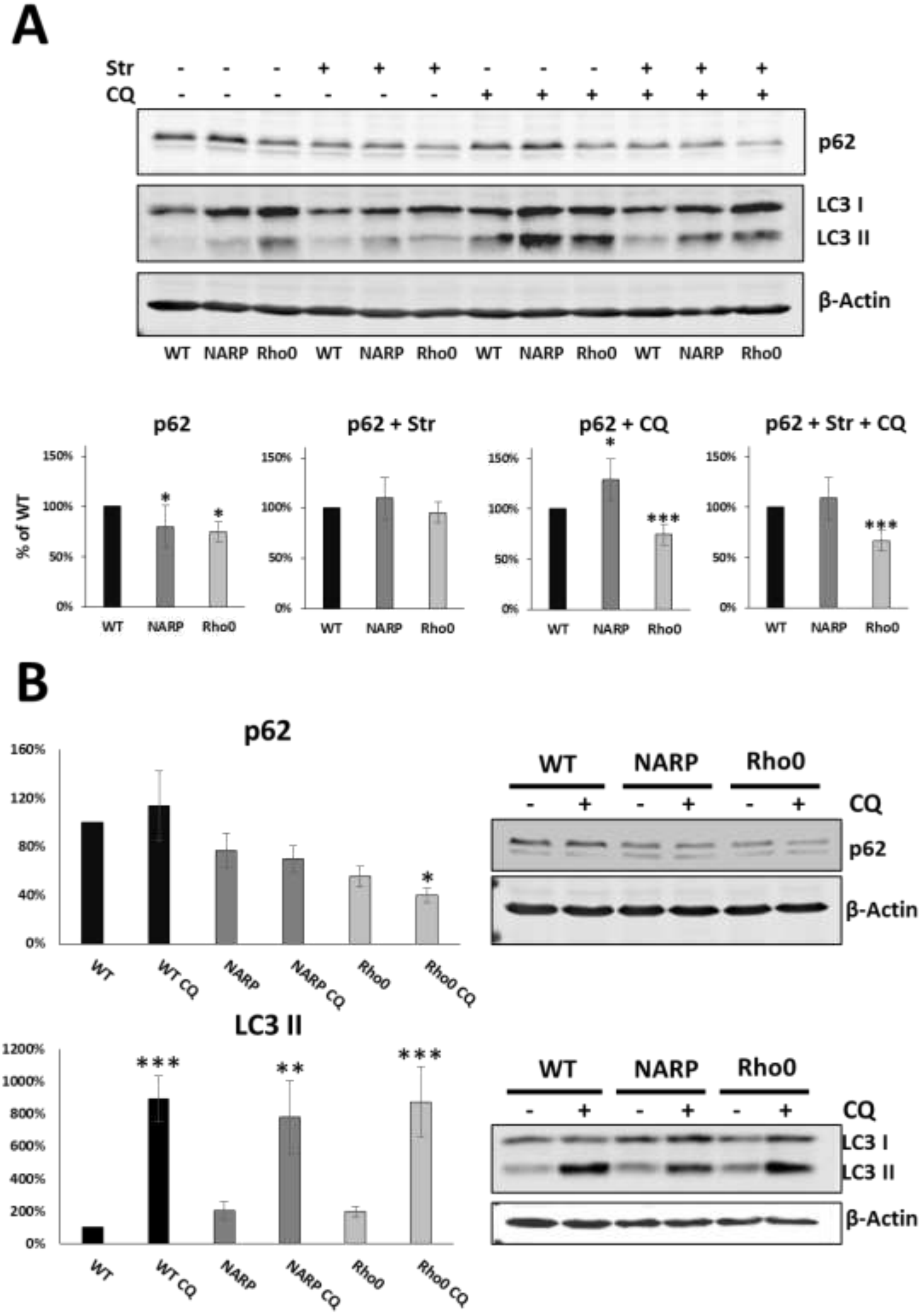


Supplementary Figure 7: Total cell lysates were analyzed by western blotting for expression of LC3 and p62 proteins. (A) Where indicated cells were treated with the autophagy inhibitor - 50 µM chloroquine (CQ) or starved (Strv) in PBS for 2 hours. Starvation caused decrease in p62 level in NARP and Rho0 cells but it had no impact on LC3 turnover. Starvation in presence of CQ caused accumulation of LC3 II but had no impact on p62 level, same as in case of CQ treatment alone. In (B) cells were treated with autophagy inhibitor – 50 µM chloroquine (CQ) for 8 hours. This treatment caused further accumulation of LC3II protein but still did not lead to the increase of p62 level, which was even lower in Rho0 cells. The values in the graphs represent the means ± SD from at least 3 independent experiments, and representative WB images are shown. β-actin was used as the loading control. The asterisks indicate values that are significantly different from the controls (WT in A or untreated WT in B): *$P < 0.05$; **$P < 0.01$, ***$P < 0.001$.

**A**

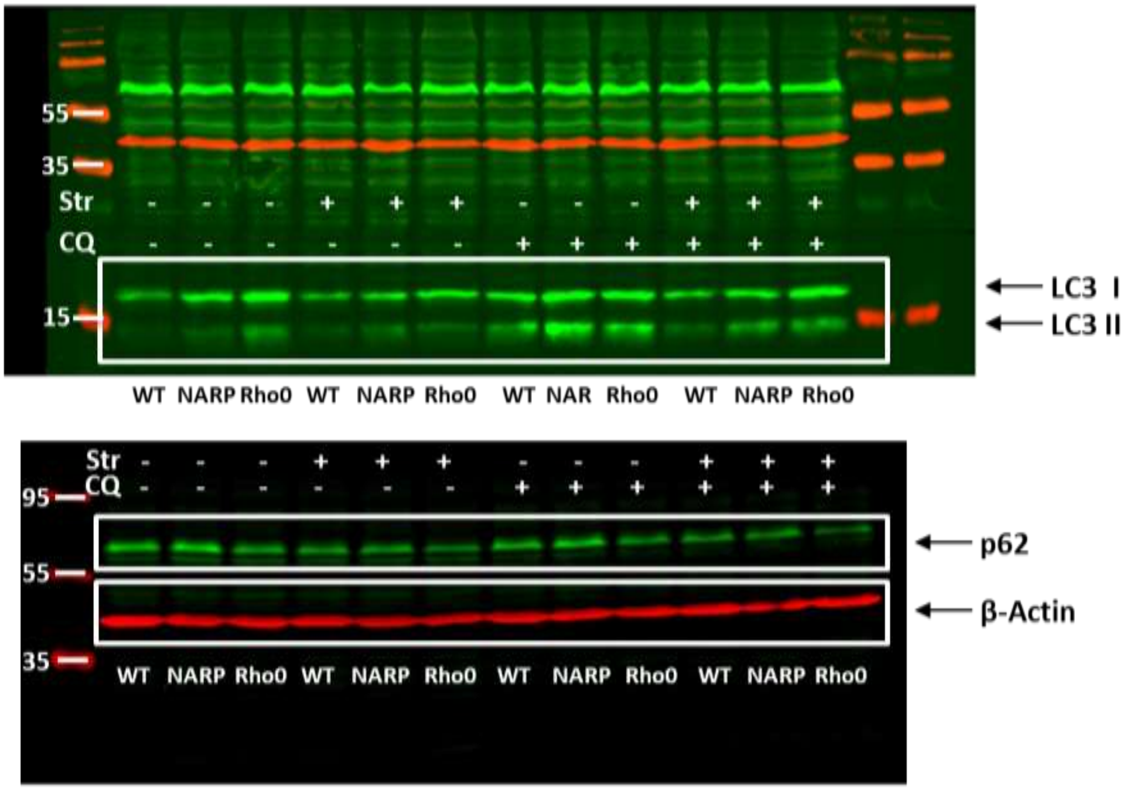


**B**

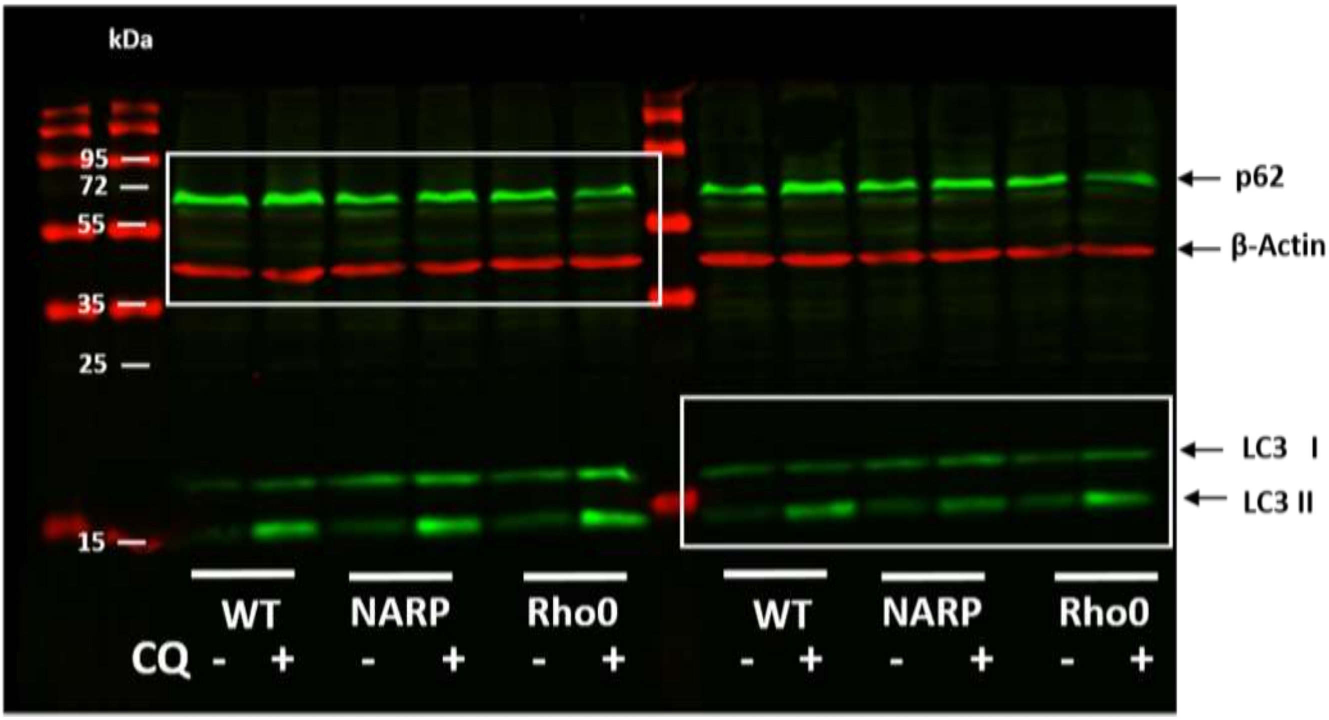


Supplementary Figure 8: full-length blots of p62, LC3 I/II and β-Actin for Supplementary Figure 7. Blots were developed with near-infrared fluorescence method (Odyssey LiCor).
